# Associations of hereditary angioedema attacks with intestinal permeability and inflammatory cytokines

**DOI:** 10.3389/fimmu.2026.1873666

**Published:** 2026-08-10

**Authors:** Xue Wang, Nan Zhou, Yuxiang Zhi

**Affiliations:** Department of Allergy & Clinical Immunology, Peking Union Medical College Hospital, Peking Union Medical College & Chinese Academy of Medical Sciences, National Clinical Research Center for Immunologic Diseases, Beijing, China

**Keywords:** gastrointestinal edema, hereditary angioedema, inflammatory cytokines, intestinal permeability, lipopolysaccharide

## Abstract

**Background:**

Hereditary angioedema (HAE) is characterized by recurrent subcutaneous and submucosal edema. Gastrointestinal involvement is common; however, the role of intestinal barrier dysfunction and its relationship with systemic inflammation in HAE remains poorly understood.

**Methods:**

This cross-sectional study included 169 patients with HAE. Circulating markers of intestinal permeability (e.g., lipopolysaccharide [LPS] and fatty acid–binding protein 2 [FABP2]) and inflammatory cytokines (e.g., interleukin [IL]-1β, IL-6, IL-10, and tumor necrosis factor-α) were measured. Associations with edema location and the HAE activity score (HAE-AS) were evaluated through univariate and multivariable regression models. Exploratory mediation and interaction analyses were performed to assess potential relationships among LPS, IL-6, and gastrointestinal edema.

**Results:**

Relative to patients without recent gastrointestinal edema, those with recent gastrointestinal edema had significantly higher serum levels of LPS and IL-6 (P = 0.030 and P = 0.031, respectively); FABP2 levels did not significantly differ. Multivariable logistic regression showed that LPS (odds ratio = 3.90, 95% CI: 1.58-10.35, P = 0.004) and IL-6 (odds ratio = 1.15, 95% CI: 1.01-1.31, P = 0.038) were independently associated with recent gastrointestinal edema. No significant mediation or interaction between LPS and IL-6 was observed. Linear regression analysis indicated that LPS was independently associated with HAE-AS (β = 1.499, P = 0.046).

**Conclusion:**

Altered intestinal permeability and inflammatory activation, particularly elevated LPS levels, are closely associated with gastrointestinal manifestations and disease activity in HAE. These findings suggest a gut–angioedema axis contributes to phenotype-specific disease expression. IL-6 may reflect concurrent inflammatory activation; the absence of elevated FABP2 suggests that gastrointestinal edema can occur without overt epithelial injury. These results support a role for the gut environment in shaping disease heterogeneity and provide a basis for further mechanistic studies.

## Introduction

Hereditary angioedema (HAE) is a rare but potentially life-threatening genetic disorder, with an estimated prevalence of 1 in 50, 000 to 1 in 100, 000 (1). It is characterized by recurrent episodes of subcutaneous or submucosal edema. Gastrointestinal involvement is common in patients with HAE, with reported rates ranging from 43% to 93%. A patient-reported survey from the United States indicated that approximately 58.0% of attacks in the preceding month involved the gastrointestinal tract (2). Gastrointestinal edema typically presents with abdominal pain, distension, vomiting, and diarrhea (1); nearly half of patients report abdominal pain as the sole symptom (3, 4). Notably, abdominal manifestations may precede cutaneous edema by several years; this pattern often leads to misdiagnosis as other acute abdominal conditions and results in unnecessary medical or surgical interventions (5, 6). Consequently, gastrointestinal edema substantially impairs quality of life in patients with HAE while increasing the risks of misdiagnosis and inappropriate treatment. Elucidation of the underlying mechanisms and identification of relevant biomarkers therefore have important clinical implications.

The pathogenesis of HAE is primarily associated with dysregulated activation of the kallikrein–kinin system and excessive bradykinin production. Based on the level and functional activity of C1 inhibitor (C1-INH), HAE is classified as C1-INH deficiency (HAE-1) or C1-INH dysfunction (HAE-2), both of which result from mutations in the *SERPING1* gene that encodes C1-INH (HAE-C1-INH) (7). C1-INH plays pivotal regulatory roles in the complement and contact systems; its deficiency or dysfunction leads to uncontrolled activation of the kallikrein–kinin cascade and overproduction of bradykinin. Bradykinin increases vascular permeability through the bradykinin B2 receptor and induces edema formation (1, 8). Additionally, some patients present with normal C1-INH levels and function (HAE with normal C1-INH, HAE-nC1-INH); in those patients, the disease is associated with variants in genes such as factor XII, angiopoietin‐1, plasminogen, kininogen, myoferlin, heparan sulfate‐glucosamine 3‐O‐sulfotransferase 6, DAB2IP, carboxypeptidase N (9–16). However, these mechanisms do not fully explain the substantial heterogeneity in anatomical distribution and edema attack severity in HAE.

The intestinal epithelium forms a selectively permeable barrier through tight junctions, which maintain immune homeostasis while limiting the translocation of microorganisms and their products across the epithelium. Structural or functional disruption of this barrier can increase intestinal permeability, triggering local and systemic inflammatory responses (17, 18). Zonulin, a key regulator of epithelial barrier permeability, has been implicated in barrier dysfunction in multiple inflammatory conditions (19). Elevated serum zonulin levels have been associated with attack frequency in HAE, suggesting that intestinal barrier dysfunction influences disease activity (19). In addition to zonulin, several circulating biomarkers may reflect intestinal permeability; however, their roles in HAE remain largely unexplored. Lipopolysaccharide (LPS), also known as endotoxin, is a major component of the outer membrane of gram-negative bacteria. When the intestinal barrier is compromised, LPS can enter the circulation and is widely regarded as an indirect marker of bacterial translocation. LPS activates Toll-like receptor 4 (TLR4) and the downstream MyD88–NF-κB signaling pathway, leading to the release of multiple proinflammatory cytokines (e.g., interleukin [IL]-6, IL-1β, and tumor necrosis factor [TNF]-α) (20–24). Additionally, experimental studies have shown that LPS can promote factor XII autoactivation and enhance kallikrein–kinin system activity, suggesting a link between gut-derived endotoxemia and bradykinin-mediated pathways in HAE (25). Fatty acid–binding protein 2 (FABP2) is another biomarker of intestinal barrier integrity. This 14–15 kDa cytosolic protein is expressed in small intestinal epithelial cells and involved in intracellular transport of long-chain fatty acids. When epithelial injury or barrier disruption occurs, FABP2 is rapidly released into the circulation; therefore, its serum level is considered a sensitive marker of intestinal epithelial damage and altered permeability (26, 27). Despite the biological relevance of these markers, data concerning intestinal permeability–related biomarkers beyond zonulin in HAE remain limited.

Emerging evidence indicates that patients with HAE exhibit systemic inflammatory activation during both remission and attack phases. This inflammatory activation state is characterized by elevated levels of proinflammatory cytokines (e.g., IL-1β, IL-6, IL-17A, TNF-α, and CXCL8), as well as the anti-inflammatory cytokine IL-10 (28–30). In other chronic inflammatory conditions, including inflammatory bowel disease, nonalcoholic fatty liver disease, and atherosclerosis, intestinal barrier disruption and LPS translocation into the circulation have been recognized as key drivers of systemic inflammation (20–23). Despite the coexistence of intestinal barrier dysfunction and systemic inflammation in HAE, the relationship between these processes remains poorly defined; integrated data linking gut permeability markers to inflammatory responses are limited. The primary objective was to determine whether circulating LPS and FABP2 levels are independently associated with HAE-C1-INH edema, particularly gastrointestinal edema. The secondary objective was to investigate the changes in inflammation in HAE-C1-INH and its interaction with circulating LPS and FABP2. By integrating gut-derived markers with inflammatory mediators, we sought to provide insights into the role of the intestinal barrier in HAE and to identify potential biomarkers associated with phenotype-specific disease expression.

## Methods

### Study design and participants

This cross-sectional study included patients with HAE-C1-INH who underwent clinical follow-up and evaluation for attacks; laboratory parameters had been recorded for all participants. Each HAE diagnosis was established according to the 2020 US Hereditary Angioedema Association Medical Advisory Board guidelines for the management of hereditary angioedema (31). Individuals were excluded if they had inflammatory bowel disease, autoimmune disorders, malignancy, hematologic disease, use of systemic immunosuppressive therapy, pregnancy, or recent antibiotic use (within 1 month prior to sampling). Demographic and clinical data were collected from the electronic medical record system and a questionnaire survey. These data included the presence of skin edema, gastrointestinal edema, and laryngeal edema during the most recent month. After exclusion of incomplete cases, 169 participants were included in the final analysis. Disease activity was assessed using the HAE activity score (HAE-AS), a short, psychometrically validated questionnaire that provides a linear measure of disease activity over the preceding 6 months (32). All participants gave written informed consent to take part in the study, and the protocol was approved by the Ethics Committee of Peking Union Medical College Hospital (HS-2402).

### Clinical and laboratory measurements

All blood samples were processed immediately after collection following a standardized institutional protocol: blood was allowed to clot, uniformly centrifuged at 3000 rpm for 15 minutes, and the separated serum aliquots were immediately transferred and stored at -80 °C until batch analysis to minimize ex vivo degradation. C1-INH antigen levels were measured using the BN™ II System (Dade Behring Marburg GmbH, Marburg, Germany). C4 concentrations were determined by immunonephelometry with the C4 reagent (29, 100 test cartridge; Beckman Coulter, CA, USA), in accordance with the manufacturers’ instructions. Serum levels of inflammatory cytokines, including IL-6, TNF-α, IL-1β, and IL-10, were quantified via commercially available enzyme-linked immunosorbent assay (ELISA) kits (FineTest, Wuhan, China) using a double-antibody sandwich method. FABP2 concentrations were measured using a Quantikine™ Human FABP2 ELISA kit (R&D Systems, Minneapolis, MN, USA). LPS levels were determined using a commercial competitive ELISA kit (Cat No. abx051541; Abbexa, Cambridge, UK) optimized for human serum, in strict accordance with the manufacturer’s protocols. Briefly, serum samples were processed using non-pyrogenic tubes, allowed to coagulate at room temperature, and centrifuged at 1000 ×*g* for 20 min at 4 °C to minimize matrix interference. The assay’s dynamic range was 0.015-1.0 EU/mL, with a minimum detection sensitivity of < 0.01 EU/mL. According to the manufacturer’s validation specifications, the intra-assay and inter-assay coefficients of variation (CV) were < 6% and < 10%, respectively. Matrix specificity and dilution linearity were validated using human serum (n=5), demonstrating acceptable recovery rates across serial dilutions (105–114% for 1:2, 95–105% for 1:4, 92–101% for 1:8, and 107–117% for 1:16 dilution). All measurements were performed in accordance with the manufacturers’ protocols.

### Statistical analysis

Continuous variables were subjected to normality assessment via the Shapiro–Wilk test. Normally distributed data are presented as mean ± standard deviation and were compared using the independent-samples t-test. Non-normally distributed data are presented as median (interquartile range) and were compared using the Wilcoxon rank-sum test. Categorical variables are presented as count (%) and were compared using the χ² test or Fisher’s exact test, as appropriate. To evaluate the monotonic relationships among laboratory variables, pairwise Spearman rank correlation coefficients were calculated among IL-10, IL-6, IL-1β, TNF-α, FABP2, LPS, C1-INH, and C4 levels.

To identify factors associated with edema location, univariate logistic regression analyses were performed for complement markers, inflammatory cytokines, and intestinal permeability–related markers. Multivariable logistic regression models were then constructed to evaluate independent associations, with adjustments for age, sex, age at onset, long-term prophylactic medication use, C1-INH level, and C4 level. To examine the association between biomarkers and disease activity, linear regression models were fitted with HAE-AS as a continuous outcome, with adjustment for the same covariates.

Given the potential interplay between intestinal permeability markers and systemic inflammatory responses, additional analyses were performed to further examine relationships among key variables. LPS and IL-6 were selected for detailed evaluation as representative markers of gut-derived endotoxemia and inflammatory activation, respectively. Mediation analysis was conducted to assess whether IL-6 mediated the association between LPS and gastrointestinal edema, implemented via the mediation package in R using nonparametric bootstrapping. Given the cross-sectional nature of this study, this analysis evaluates potential statistical pathways and produces associational estimates only, as the temporal ordering of variables cannot be verified. Interaction analysis was performed by including an LPS × IL-6 interaction term in the multivariable logistic regression model.

To evaluate the robustness of our primary findings, two sensitivity analyses were conducted using the fully adjusted multivariable models. First, to address potential reverse causation, an analysis was restricted to patients who remained completely attack-free at any site within 30 days prior to blood sampling. Second, to eliminate pharmacological confounding, another analysis was restricted to patients who were not receiving long-term prophylaxis; in this subpopulation model, prophylactic medication use was removed from the covariates while adjustments for age, sex, age at onset, C1-INH, and C4 levels were maintained.

To evaluate the clinical discriminative value of circulating LPS for recent gastrointestinal attacks, receiver operating characteristic (ROC) curves were constructed using the pROC package based on the fully adjusted multivariable models, with probabilities corrected via Firth’s penalized likelihood method (logistf package).

Two-sided P-values < 0.05 were considered statistically significant. All analyses were performed using R software (version 4.5.1; R Foundation for Statistical Computing, Vienna, Austria).

## Results

In total, 169 patients were included, of whom 127 (75.1%) were female. The mean age was 39 years, and the mean age at disease onset was 20.59 ± 11.50 years. A positive family history was reported by 75.7% of patients. Nearly all patients had experienced skin edema (94.7%); high proportions reported gastrointestinal (76.9%) and laryngeal edema (52.1%). Overall, 58.0% of patients had at least one emergency department visit due to edema attacks. In the month preceding sampling, 69.2% of patients reported skin edema, 39.6% had gastrointestinal edema, and 18.9% experienced laryngeal edema. The mean annual number of life-threatening edema episodes per patient was 0.13 ± 0.34. More than half of the patients (55.0%) did not receive long-term prophylaxis, whereas 41.4% received danazol and 3.0% received lanadelumab. None of the patients in this cohort received short-term prophylaxis or treatment for acute attacks with C1-INH concentrate or icatibant. The mean HAE-AS was 4.99 ± 3.83. C1-INH and complement C4 levels were considerably reduced, with median [interquartile range] values of 0.07 [0.05, 0.09] and 0.05 [0.03, 0.08], respectively. Circulating markers related to inflammation and intestinal permeability, including IL-10, IL-6, IL-1β, TNF-α, FABP2, and LPS, are summarized in **Table 1**.

**Table 1:** Demographic characteristics of the study participants.

Variable	Level	Overall
Samples		169
Sex	Female	127 (75.1)
	Male	42 (24.9)
Age (y)		39 ± 13.31
Onset age (y)		20.59 ± 11.50
Skin edema		160 (94.7)
Gastrointestinal edema		130 (76.9)
Laryngeal edema		88 (52.1)
Recent skin edema		117 (69.2)
Recent gastrointestinal edema		67 (39.6)
Recent laryngeal edema		32 (18.9)
Number of life-threatening edema episodes per year		0.13 ± 0.34
Long-term prophylaxis	No medication administered	93 (55.0)
	Lanadelumab	5 (3.0)
	Danazol	70 (41.4)
Emergency department visit		98 (58.0)
Family history		128 (75.7)
HAE-AS		4.99 ± 3.83
C1-INH		0.07 [0.05, 0.09]
C4		0.05 [0.03, 0.08]
IL-10		8.02 [4.84, 23.92]
IL-6		2.58 [1.51, 4.91]
IL-1β		2.36 [1.41, 7.02]
FABP2		1597.40 [1071.06, 2338.00]
TNF-α		24.21 [11.27, 151.56]
LPS		1.23 [1.05, 1.51]

Data are presented as mean ± standard deviation, median [interquartile range], or n (%). The units are ng/mL for FABP2, EU/mL for LPS, and pg/mL for IL-10, IL-6, IL-1β, and TNF-α.

Patients with gastrointestinal edema had significantly lower C1-INH levels relative to those without gastrointestinal involvement (0.07 [0.05–0.09] vs 0.08 [0.06–0.12], P = 0.038), along with higher circulating LPS levels (1.23 [1.07–1.53] vs 1.17 [1.00–1.38], P = 0.049). No significant differences were observed for other inflammatory markers (**Table 2**). Patients with recent gastrointestinal edema (in the past month) showed lower C1-INH (P = 0.047) and C4 levels (P = 0.032), as well as higher IL-6 (P = 0.031) and LPS levels (P = 0.030), relative to patients without recent episodes (**Table 2**). In contrast, skin and laryngeal edema phenotypes showed weaker associations with markers of intestinal permeability and inflammation (**Supplementary Table 1**). Furthermore, patients with skin edema in the past month had lower C4 levels (P = 0.030) and higher IL-6 levels (P = 0.046) relative to patients without recent skin edema (**Supplementary Table 1**). Similarly, patients with recent laryngeal edema had lower C4 levels than those without (P = 0.014); other markers did not significantly differ (**Supplementary Table 1**). Finally, patients with life-threatening edema episodes in the past month showed reduced C1-INH (P = 0.025) and C4 levels (P = 0.013), whereas inflammatory and intestinal permeability markers did not significantly differ (**Supplementary Table 1**). Spearman correlation analysis revealed robust mutual correlations among systemic inflammatory cytokines and a significant positive linkage between C1-INH and C4 levels. However, circulating LPS and FABP2 levels did not demonstrate significant monotonic correlations with either inflammatory cytokines or complement pathways (**Supplementary Figure 1**).

**Table 2:** Intestinal permeability and inflammatory markers in patients with HAE, according to gastrointestinal edema status.

Variable	Level	Without gastrointestinal edema	With gastrointestinal edema	*p*-value	Without gastrointestinal edema in past month	With gastrointestinal edema in past month	*p*-value
Sex	Female	25 (73.5)	98 (75.4)	1	76 (76.8)	49 (73.1)	0.727
	Male	9 (26.5)	32 (24.6)		23 (23.2)	18 (26.9)	
Age		37.65 (12.91)	39.68 (13.29)	0.426	37.93 (11.88)	40.60 (15.18)	0.207
C1-INH		0.08 [0.06, 0.12]	0.07 [0.05, 0.09]	0.038	0.07 [0.06, 0.10]	0.06 [0.05, 0.08]	0.047
C4		0.06 [0.03, 0.07]	0.05 [0.02, 0.08]	0.852	0.06 [0.03, 0.08]	0.05 [0.02, 0.07]	0.032
IL-10		6.02 [4.27, 24.57]	8.20 [5.48, 23.41]	0.066	7.84 [4.63, 20.68]	7.93 [5.67, 24.05]	0.471
IL-6		2.16 [1.36, 4.41]	2.61 [1.57, 4.99]	0.254	2.17 [1.28, 4.10]	2.81 [1.83, 5.54]	0.031
IL-1β		1.87 [1.19, 6.66]	2.44 [1.50, 6.52]	0.118	2.18 [1.42, 5.94]	2.40 [1.44, 6.02]	0.557
FABP2		1778.08 [1198.51, 2301.04]	1576.18 [1069.51, 2340.55]	0.666	1572.96 [1078.67, 2298.04]	1711.09 [1084.36, 2431.01]	0.653
TNF-α		22.03 [11.77, 193.96]	26.94 [10.75, 150.39]	0.794	24.21 [10.08, 144.93]	25.37 [12.27, 209.65]	0.604
LPS		1.17 [1.00, 1.38]	1.23 [1.07, 1.53]	0.049	1.19 [1.02, 1.42]	1.27 [1.07, 1.70]	0.030

*Discrepancies between the subgroup sums and the total cohort are due to the fact that some patients had missing C1-INH and C4 levels and were thus excluded from these analyses.

**Figure 1:**
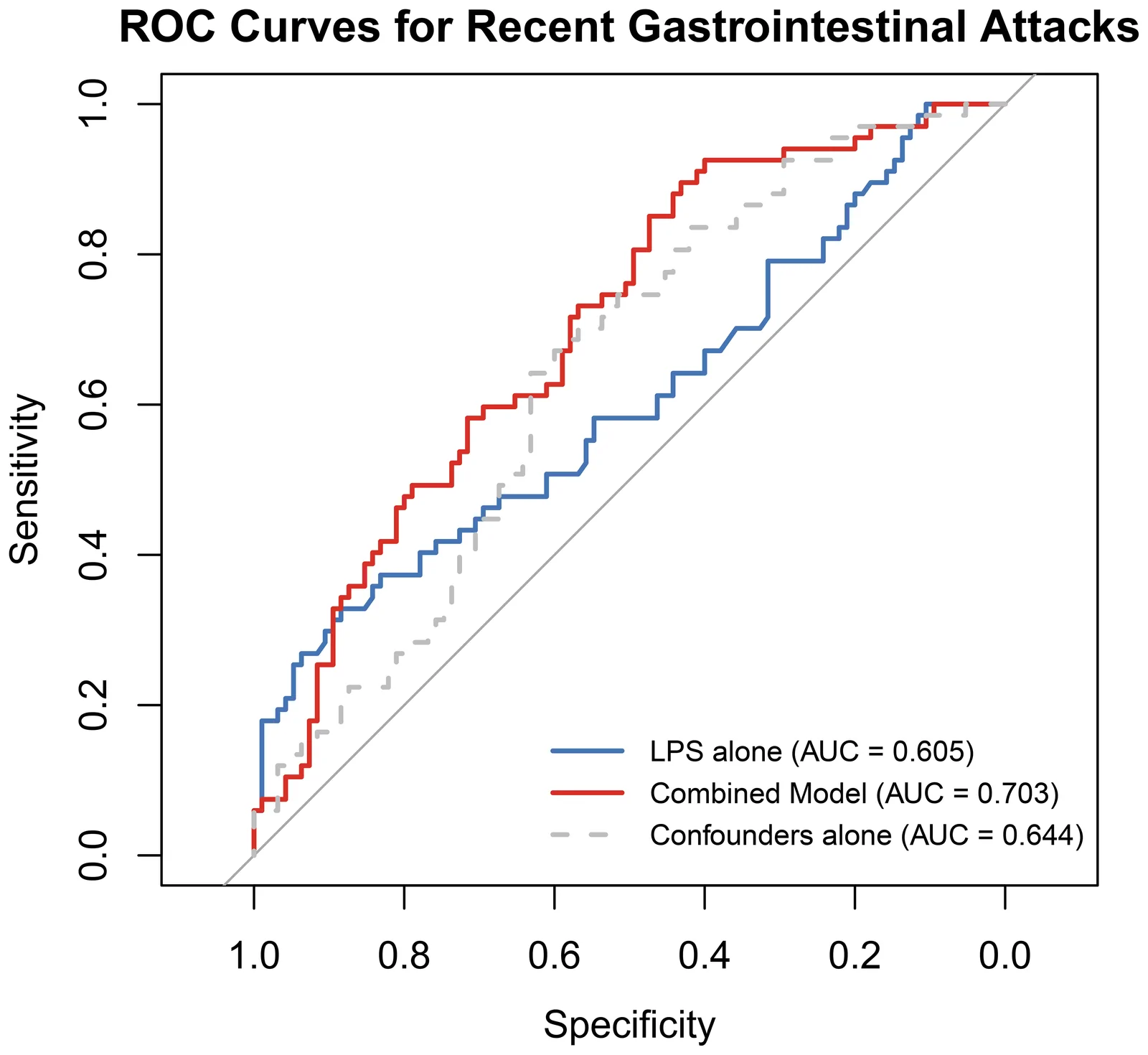
ROC curves for recent gastrointestinal attacks.

Univariate logistic regression identified IL-6 and LPS as factors significantly associated with recent gastrointestinal edema (**Table 3**). Higher IL-6 levels were associated with increased odds of recent gastrointestinal involvement (odds ratio [OR] 1.153, 95% confidence interval [CI] 1.031–1.308, P = 0.018); elevated LPS levels also were associated with recent gastrointestinal involvement (OR 3.580, 95% CI 1.562–8.709, P = 0.003). Other markers, including IL-10, IL-1β, FABP2, and TNF-α, showed no significant associations. In multivariable analysis, both IL-6 and LPS remained independently associated with recent gastrointestinal edema (**Table 3**). IL-6 showed a modest but significant effect (OR 1.145, 95% CI 1.014–1.311, P = 0.038), whereas LPS demonstrated a stronger association (OR 3.9, 95% CI 1.581–10.354, P = 0.004). No significant associations were observed for the remaining variables (**Table 3**). Mediation analysis demonstrated that the total effect of LPS on recent gastrointestinal edema was significant (total effect = 0.21, P < 0.001). However, IL-6 did not show a significant mediating effect (average causal mediation effect = 0.011, P = 0.54), with a mediation proportion of approximately 5% (P = 0.54) (**Supplementary Figure 2**). These findings suggest that IL-6 does not significantly mediate the association between LPS and gastrointestinal edema; the observed effect of LPS is primarily direct. Additionally, no significant interaction between LPS and IL-6 was detected (P = 0.465), indicating no evidence of effect modification between these markers (**Supplementary Table 2**). Furthermore, ROC analysis based on the multivariable models demonstrated that incorporating LPS into the baseline model (comprising conventional clinical confounders and complement markers) increased the AUC from 0.644 (95% CI: 0.560–0.729) to 0.703 (95% CI: 0.623–0.783) (**Figure 1**).

**Table 3:** Univariate and multivariable logistic regression analyses of laboratory parameters associated with recent gastrointestinal edema.

	Univariate logistic regression			Multivariate logistic regression		
Variable	OR	95% CI	*p*-value	OR	95% CI	*p*-value
IL-10	1.001	0.998-1.003	0.5739	1.001	0.999-1.003	0.4628
IL-6	1.153	1.031-1.308	0.0181	1.145	1.014-1.311	0.0376
IL-1β	1.001	0.998-1.005	0.4379	1.001	0.998-1.005	0.3806
FABP2	1.000	1.000-1.000	0.6582	1.000	1.000-1.000	0.1545
TNF-α	1.000	1.000-1.001	0.3984	1.000	1.000-1.001	0.1574
LPS	3.580	1.562-8.709	0.0034	3.900	1.581-10.354	0.0042

Linear regression analyses were performed to evaluate associations between laboratory biomarkers and disease activity, as measured by HAE-AS. In univariate analysis, IL-6 was positively associated with HAE-AS (β = 0.245, P = 0.016) (**Table 4**). In multivariable analysis with adjustment for potential confounders, LPS remained independently associated with higher HAE-AS values (β = 1.499, P = 0.046). IL-6 and IL-1β showed trends toward positive associations with disease activity; however, these relationships were not statistically significant (**Table 4**).

**Table 4:** Univariate and multivariable linear regression analyses of factors associated with HAE disease activity (HAE-AS).

	Univariate analysis		Multivariate analysis	
Variable	β	p-value	β	p-value
C1-INH	-0.486	0.841	-1.570	0.497
C4	-0.016	0.782	-0.001	0.992
IL-10	0.002	0.213	0.003	0.157
IL-6	0.245	0.016	0.172	0.085
IL-1β	0.005	0.094	0.005	0.072
FABP2	0.0002	0.434	0.0001	0.741
TNF-α	0.001	0.267	0.001	0.311
LPS	0.863	0.256	1.499	0.046

Two sensitivity analyses were performed to test the robustness of our primary findings (**Supplementary Table 3** and **S4**). First, we restricted the model to patients who were completely attack-free within 30 days prior to blood sampling (**Supplementary Table 3**). In this subpopulation, the independent associations of LPS and IL-6 with gastrointestinal manifestations and HAE-AS were no longer statistically significant. Second, to eliminate pharmacological confounding, the multivariable model was restricted to treatment-naive patients (**Supplementary Table 4**). Within this untreated cohort, the associations of LPS and IL-6 with recent gastrointestinal edema did not reach statistical significance, though the point estimate for LPS remained elevated (OR = 2.087, 95% CI: 0.658–7.060, *p* = 0.219).

## Discussion

HAE exhibits considerable clinical heterogeneity, and the underlying mechanisms remain incompletely understood (33). In the present study, we systematically evaluated intestinal permeability–related markers and inflammatory profiles across various clinical phenotypes of HAE and examined their associations with disease activity. Markers of gut barrier dysfunction, particularly LPS, were significantly elevated in patients with gastrointestinal involvement and were independently associated with recent gastrointestinal edema. In parallel, IL-6 showed a modest but significant association with gastrointestinal attacks; other inflammatory cytokines did not demonstrate consistent associations. Changes in intestinal permeability and inflammatory markers were more pronounced among patients with gastrointestinal manifestations than among patients with skin or laryngeal edema. Additionally, C1-INH and C4 levels were consistently lower in patients with recent or more severe attacks, reflecting their acute consumption during recent edematous episodes. Collectively, these findings suggest associations of intestinal barrier dysfunction with systemic inflammation and potential gastrointestinal edema in HAE; they highlight a previously underrecognized role for the gut–vascular axis in this disorder.

The association between elevated LPS levels and gastrointestinal edema in HAE suggests a connection between intestinal barrier dysfunction and gastrointestinal manifestations. Circulating LPS is widely regarded as an indirect indicator of impaired intestinal barrier integrity and translocation of bacterial products, a phenomenon documented in multiple disorders. In inflammatory bowel disease, LPS has been shown to exacerbate mucosal inflammation by downregulating tight junction proteins (e.g., occludin and claudin-1) and activating the TLR4/NF-κB signaling pathway (34). Similarly, increased fecal LPS levels have been detected in patients with diarrhea-predominant irritable bowel syndrome and demonstrated associations with symptom severity, supporting a role for endotoxin in gut-related disease manifestations (34). Our previous work demonstrated that patients with recent HAE attacks, particularly those with abdominal symptoms, showed significantly reduced microbial richness and diversity (35). These patients also displayed alterations in gut microbiota composition, including Firmicutes depletion and Proteobacteria enrichment (35). Such microbial changes have been linked to impaired epithelial barrier integrity (35), which may promote endotoxin production and translocation. Considered along with the present findings of elevated circulating LPS—especially in patients with gastrointestinal edema—these results support the presence of a disrupted gut barrier and increased exposure to microbial products in HAE patients with gastrointestinal manifestations.

Causal pathways could not be fully established in the present study, but there is growing evidence that microbial products such as LPS may influence vascular permeability in HAE. Experimental studies have shown that LPS can interact with high-molecular-weight kininogen and activate the contact system (36). Furthermore, LPS has been reported to directly promote factor XII activation *in vitro (*37), providing a potential mechanistic link between endotoxemia and the kallikrein–kinin system. Although these pathways were not directly assessed, the independent association between LPS and recent gastrointestinal edema identified in our study supports the hypothesis that gut-derived endotoxemia can contribute to disease expression by modulating vascular permeability.

Our findings, together with those of a recent study, suggest that intestinal permeability plays an important role in edema pathogenesis among patients with HAE. Kural and colleagues reported that elevated serum zonulin levels were independently associated with attack frequency in HAE (19). Building on this observation, the present study demonstrated that LPS is associated with the gastrointestinal edema phenotype and disease activity, providing additional support for a gut–angioedema axis. We also examined the potential role of inflammatory mediators within this framework. Although both IL-6 and LPS were associated with gastrointestinal edema, mediation analysis did not reveal a significant mediating effect for IL-6. This result suggests that the relationship between LPS and gastrointestinal involvement is not primarily explained by IL-6–driven systemic inflammation. An important alternative explanation for the observed association between LPS and gastrointestinal edema is reverse causation. Gastrointestinal edema itself may impair intestinal barrier integrity through bowel wall distension, local ischemia, or mucosal dysfunction, thereby promoting translocation of bacterial products and increasing circulating LPS levels. To partially address this possibility, we performed sensitivity analyses after excluding patients who had experienced any edema attack within 30 days before blood sampling. In these analyses, the associations between LPS, IL-6, and gastrointestinal edema were attenuated and no longer statistically significant. These findings suggest that elevated circulating LPS may be influenced by recent disease activity and may reflect attack-related alterations in gut barrier function rather than serving as a stable marker of lifetime gastrointestinal involvement. Therefore, although our primary analyses support an association between intestinal permeability and gastrointestinal manifestations in HAE, the temporal relationship remains uncertain and reverse causation cannot be excluded.

FABP2 is widely regarded as a sensitive circulating marker of intestinal epithelial injury. Elevated FABP2 levels have been reported in conditions associated with overt mucosal damage or ischemia, such as sepsis, septic shock, mesenteric ischemia, and strangulated intestinal obstruction (38–40), as well as metabolic disorders associated with intestinal barrier impairment (41–43). Authors of previous studies have proposed that intestinal injury exists along a continuum ranging from functional disturbance to overt structural damage; FABP2 elevation is typically observed at more advanced stages characterized by epithelial disruption (44). In the present study, FABP2 levels did not significantly differ according to edema phenotype or recent attack status. This finding suggests that gastrointestinal involvement in HAE does not involve substantial acute epithelial structural injury but may instead reflect functional alterations in intestinal barrier or vascular permeability. This intriguing dissociation—characterized by elevated circulating LPS in the presence of normal FABP2 levels—provides pivotal mechanistic insight into the pathophysiology of gastrointestinal HAE. It strongly implies that the gut barrier impairment is driven by functional vascular hyperpermeability, rather than overt epithelial structural damage. Furthermore, our sensitivity analyses demonstrated that the independent associations of LPS and IL-6 with gastrointestinal manifestations were attenuated and no longer statistically significant after excluding patients with recent attacks. This temporal volatility further supports the concept that the elevated circulating LPS reflects transient, attack-related functional “leakiness” of the gut barrier rather than a stable, permanent structural defect.

Additionally, we examined the role of inflammatory markers in HAE-related edema. IL-6 is a key proinflammatory cytokine involved in the regulation of vascular permeability (45). In the present study, IL-6 was positively associated with recent gastrointestinal edema in multivariable analysis and showed a tendency toward association with disease activity, suggesting a role for inflammatory activation in HAE disease expression. Notably, IL-6 is induced by LPS exposure through activation of the TLR4–NF-κB signaling pathway (46), which provides a biologically plausible link between intestinal permeability and systemic inflammation. In this context, the observed associations of LPS and IL-6 with gastrointestinal edema support the hypothesis that gut-derived endotoxemia at least partially contributes to disease activity through downstream inflammatory responses. However, mediation analysis did not demonstrate a significant indirect effect of IL-6 on the relationship between LPS and gastrointestinal edema; no significant interaction between LPS and IL-6 was identified. These findings suggest that, although both LPS and IL-6 are associated with gastrointestinal involvement, their effects may be independent or only partially overlapping, rather than the outcome of a simple linear pathway.

This study has several strengths. We evaluated markers of intestinal permeability together with inflammatory mediators within the same cohort, enabling integrated assessment of their relationships. Additionally, the inclusion of multiple markers (e.g., LPS and FABP2), rather than reliance on a single indicator, provided a broader characterization of intestinal barrier status in HAE. Another strength is the analysis of edema sites and recent attack status. Despite the unpredictability of HAE attacks and the difficulty of obtaining samples during acute episodes in clinical practice, we included patients who had experienced edema within 1 month prior to sampling. Although this approach is indirect, it may better approximate biomarker changes associated with recent disease activity and provide insight into the biological characteristics of HAE before and after an attack. Several limitations should also be considered. Firstly, the cross-sectional design precludes determination of temporal or causal relationships. Although we observed an increase in LPS, edema-induced intestinal wall distension or ischemia itself may impair barrier integrity and cause LPS translocation as a consequence—rather than a driver—of attacks. Secondly, mediation and interaction analyses were performed, but their cross-sectional nature yields associational estimates only and prevents the verification of temporal ordering. Consequently, these analytical findings cannot imply definitive causal pathways, and the absence of experimental data prevents further confirmation of the underlying biological mechanisms. Thirdly, the results of this cross-sectional study may be subject to confounding factors. Variables such as pain, stress, vomiting, and medication use during episodes of gastrointestinal edema might influence intestinal permeability and inflammatory markers; their effects were not fully assessed. Fourthly, this study did not include a healthy control group, and all biomarker comparisons were internal to the HAE cohort. Consequently, no inference regarding the absolute elevation of these biomarkers above normal physiological thresholds can be made from this dataset. Fifthly, functional C1-INH activity was not systematically evaluated due to the lack of routine availability of this assay at our clinical center, though all diagnoses were firmly established based on severe C1-INH antigen deficiency and clinical presentation. Lastly, due to the marked overlap in the distributions of LPS between patients with and without gastrointestinal edema, we could not establish clinically useful cut-off values for individual diagnosis. These biomarkers currently serve as indicators of population-level biological associations rather than precise clinical diagnostic thresholds.

## Conclusion

This study provides evidence that alterations in intestinal permeability and inflammatory activity are associated with clinical manifestations of HAE, particularly gastrointestinal involvement. Among the markers evaluated, LPS showed consistent associations with recent gastrointestinal edema and disease activity, whereas IL-6 may reflect concurrent inflammatory activation. In contrast, FABP2 did not differ across clinical phenotypes, suggesting that gastrointestinal edema in HAE can occur without overt epithelial injury. Collectively, these findings support a role for the gut environment in shaping disease expression among patients with HAE. Although the underlying mechanisms require further investigation, these results indicate that markers of intestinal permeability can provide additional insights regarding disease activity and heterogeneity.

## Data Availability

The raw data supporting the conclusions of this article will be made available by the authors, without undue reservation.
