# Supplementary material for: Associations of hereditary angioedema attacks with intestinal permeability and inflammatory cytokines
Source: Front Immunol. 2026 Aug 10;17:1873666. doi: 10.3389/fimmu.2026.1873666 (PMC13500300; doi:10.3389/fimmu.2026.1873666)
Supplement: Supplementary file 1 [file Table1.docx]

**Supplementary Material**

Figure S1. Spearman correlation matrix among circulating lipopolysaccharide, intestinal barrier markers, systemic inflammatory cytokines, and complement components.

Figure S2. Mediation effect of IL-6 on the association between LPS and recent gastrointestinal edema

Table S1. Intestinal permeability and inflammatory markers in patients with HAE according to clinical phenotype

|  | level | Without skin edema | With skin edema | *p*-value | Without laryngeal edema | With laryngeal edema | *p*-value | Without skin edema in past month | With skin edema in past month | *p*-value |
| --- | --- | --- | --- | --- | --- | --- | --- | --- | --- | --- |
| Sex | Female | 2 (50.0) | 121 (75.6) | 0.559 | 58 (76.3) | 65 (73.9) | 0.857 | 37 (78.7) | 86 (73.5) | 0.618 |
|  | Male | 2 (50.0) | 39 (24.4) |  | 18 (23.7) | 23 (26.1) |  | 10 (21.3) | 31 (26.5) |  |
| Age |  | 30.50 (15.15) | 39.48 (13.13) | 0.180 | 37.95 (13.75) | 40.39 (12.67) | 0.239 | 41.98 (12.14) | 38.16 (13.50) | 0.094 |
| C1-INH |  | 0.08 [0.06, 0.14] | 0.07 [0.05, 0.09] | 0.529 | 0.06 [0.05, 0.10] | 0.07 [0.06, 0.08] | 0.707 | 0.07 [0.06, 0.11] | 0.07 [0.05, 0.09] | 0.083 |
| C4 |  | 0.07 [0.05, 0.07] | 0.05 [0.03, 0.08] | 0.791 | 0.06 [0.02, 0.08] | 0.05 [0.03, 0.08] | 0.695 | 0.06 [0.04, 0.08] | 0.05 [0.02, 0.07] | 0.030 |
| IL-10 |  | 6.76 [2.89, 23.71] | 7.97 [4.94, 23.99] | 0.424 | 7.66 [4.61, 21.73] | 8.19 [6.16, 23.99] | 0.288 | 9.61 [4.75, 53.16] | 7.72 [4.97, 19.50] | 0.494 |
| IL-6 |  | 3.16 [1.88, 5.75] | 2.56 [1.46, 4.95] | 0.522 | 2.60 [1.54, 5.01] | 2.17 [1.28, 4.80] | 0.364 | 2.17 [1.00, 3.52] | 2.62 [1.56, 5.42] | 0.046 |
| IL-1β |  | 1.91 [1.85, 5.99] | 2.39 [1.41, 7.02] | 0.991 | 2.21 [1.44, 7.02] | 2.42 [1.41, 5.78] | 0.72 | 2.81 [1.45, 8.84] | 2.25 [1.42, 4.87] | 0.551 |
| FABP2 |  | 2198.99 [1906.50, 2342.07] | 1604.49 [1084.03, 2338.00] | 0.343 | 1536.37 [1070.55, 2201.97] | 1726.77 [1186.54, 2679.20] | 0.12 | 1978.86 [1300.92, 2338.00] | 1534.31 [1057.73, 2405.04] | 0.21 |
| TNF-α |  | 20.39 [13.19, 133.55] | 26.45 [11.33, 155.55] | 0.757 | 28.28 [11.74, 188.60] | 23.92 [11.27, 127.48] | 0.846 | 26.21 [13.85, 330.07] | 24.21 [11.27, 82.87] | 0.495 |
| LPS |  | 0.77 [0.67, 0.90] | 1.23 [1.06, 1.51] | 0.013 | 1.23 [1.04, 1.57] | 1.21 [1.04, 1.46] | 0.695 | 1.18 [1.07, 1.37] | 1.25 [1.03, 1.53] | 0.317 |

Table S1. Continued

|  | level | Without laryngeal edema in past month | With laryngeal edema in past month | *p*-value | Without life-threatening edema in the past month | With life-threatening edema in the past month | *p*-value |
| --- | --- | --- | --- | --- | --- | --- | --- |
| Sex | Female | 102 (76.1) | 23 (71.9) | 0.786 | 89 (76.1) | 36 (73.5) | 0.875 |
|  | Male | 32 (23.9) | 9 (28.1) |  | 28 (23.9) | 13 (26.5) |  |
| Age |  | 39.01 (12.81) | 39.00 (15.55) | 0.998 | 39.89 (13.30) | 36.90 (13.30) | 0.188 |
| C1-INH |  | 0.07 [0.05, 0.09] | 0.06 [0.05, 0.08] | 0.313 | 0.07 [0.06, 0.10] | 0.06 [0.05, 0.08] | 0.025 |
| C4 |  | 0.06 [0.04, 0.08] | 0.03 [0.02, 0.05] | 0.014 | 0.06 [0.04, 0.08] | 0.04 [0.02, 0.06] | 0.013 |
| IL-10 |  | 7.84 [4.69, 25.74] | 8.37 [6.14, 12.90] | 0.609 | 7.84 [4.72, 23.92] | 8.07 [5.01, 21.87] | 0.715 |
| IL-6 |  | 2.56 [1.32, 4.76] | 2.76 [1.84, 5.01] | 0.375 | 2.55 [1.24, 4.43] | 2.64 [1.74, 5.42] | 0.199 |
| IL-1β |  | 2.30 [1.41, 7.02] | 2.39 [1.52, 4.13] | 0.992 | 2.25 [1.42, 4.49] | 2.39 [1.41, 8.53] | 0.807 |
| FABP2 |  | 1576.18 [1069.51, 2281.34] | 1839.68 [1178.61, 3402.11] | 0.113 | 1544.88 [1068.99, 2311.18] | 1742.44 [1203.25, 2837.57] | 0.146 |
| TNF-α |  | 26.45 [10.88, 215.08] | 23.35 [11.66, 91.95] | 0.896 | 26.21 [10.57, 151.56] | 23.92 [12.00, 142.96] | 0.755 |
| LPS |  | 1.23 [1.06, 1.51] | 1.21 [0.99, 1.53] | 0.684 | 1.19 [1.06, 1.48] | 1.35 [1.00, 1.58] | 0.358 |

Table S2. Interaction effects between LPS and IL‑6

|  | Estimate | Std. Error | z value | p-value |
| --- | --- | --- | --- | --- |
| (Intercept) | -3.11704 | 1.24899 | -2.496 | 0.0126 |
| LPS | 1.73948 | 0.74194 | 2.344 | 0.0191 |
| IL-6 | 0.24951 | 0.18899 | 1.32 | 0.1868 |
| C1-INH | -3.08528 | 1.94731 | -1.584 | 0.1131 |
| C4 | -6.64387 | 5.19092 | -1.28 | 0.2006 |
| Lanadelumab | -0.05803 | 1.17274 | -0.049 | 0.9605 |
| Danazol | -0.23148 | 0.38848 | -0.596 | 0.5513 |
| Male | 0.50102 | 0.41554 | 1.206 | 0.2279 |
| Age | 0.03258 | 0.01584 | 2.057 | 0.0397 |
| Onset age | -0.02635 | 0.01728 | -1.525 | 0.1273 |
| LPS:IL6 | -0.09622 | 0.13165 | -0.731 | 0.4649 |

Table S3. Multivariable logistic and linear regression analyses restricted to patients without any edema attacks within 30 days prior to blood sampling.

|  | Gastrointestinal edema | | | Laryngeal edema | | | HAE-AS | |
| --- | --- | --- | --- | --- | --- | --- | --- | --- |
| Variable | OR | 95% CI | p-value | OR | 95% CI | p-value | β | p-value |
| IL-10 | 1.121 | 1.026-1.366 | 0.0869 | 0.018 | 1-1.051 | 0.1482 | -0.001 | 0.263 |
| IL-6 | 1.183 | 0.661-2.419 | 0.5859 | -0.639 | 0.182-1.065 | 0.149 | 0.001 | 0.947 |
| IL-1β | 1.255 | 1.059-1.786 | 0.0486 | 0.038 | 0.994-1.115 | 0.1716 | -0.002 | 0.271 |
| FABP2 | 1 | 0.998-1.001 | 0.5611 | 0 | 0.999-1.002 | 0.5928 | 0 | 0.314 |
| TNF-α | 1.012 | 1.003-1.032 | 0.0596 | 0.005 | 1-1.013 | 0.1323 | 0 | 0.199 |
| LPS | 0.709 | 0.002-216.274 | 0.9008 | 0.597 | 0.06-57.829 | 0.7254 | -0.01 | 0.942 |

Table S4. Multivariable logistic and linear regression analyses restricted to patients not receiving long-term prophylaxis

|  | Recent gastrointestinal edema | | | Recent laryngeal edema | | | HAE-AS | |
| --- | --- | --- | --- | --- | --- | --- | --- | --- |
| Variable | OR | 95% CI | p-value | OR | 95% CI | p-value | β | p-value |
| IL-10 | 1.001 | 0.996-1.005 | 0.799 | 0.998 | 0.991-1.003 | 0.537 | 0 | 0.99 |
| IL-6 | 1.101 | 0.941-1.299 | 0.234 | 0.991 | 0.827-1.173 | 0.915 | -0.041 | 0.772 |
| IL-1β | 1.002 | 0.996-1.009 | 0.517 | 1.002 | 0.995-1.008 | 0.482 | 0.004 | 0.411 |
| FABP2 | 1 | 0.999-1 | 0.142 | 1 | 1-1.001 | 0.018 | 0 | 0.746 |
| TNF-α | 1.001 | 1-1.002 | 0.303 | 1 | 0.999-1.001 | 0.784 | 0 | 0.781 |
| LPS | 2.087 | 0.658-7.06 | 0.219 | 0.778 | 0.216-2.644 | 0.691 | 0.776 | 0.441 |
